# Zwitterionic Polymer Coated and Aptamer Functionalized Flexible Micro-Electrode Arrays for In Vivo Cocaine Sensing and Electrophysiology

**DOI:** 10.3390/mi14020323

**Published:** 2023-01-27

**Authors:** Bingchen Wu, Elisa Castagnola, Xinyan Tracy Cui

**Affiliations:** 1 Department of Bioengineering, University of Pittsburgh, Pittsburgh, PA 15213, USA; 2 Center for the Neural Basis of Cognition, Pittsburgh, PA 15213, USA; 3 Department of Biomedical Engineering, Louisiana Tech University, Ruston, LA 71272, USA; 4 McGowan Institute for Regenerative Medicine, Pittsburgh, PA 15219, USA

**Keywords:** aptasensor, zwitterionic polymer, in vivo cocaine sensing, electrophysiology

## Abstract

The number of people aged 12 years and older using illicit drugs reached 59.3 million in 2020, among which 5.2 million are cocaine users based on the national data. In order to fully understand cocaine addiction and develop effective therapies, a tool is needed to reliably measure real-time cocaine concentration and neural activity in different regions of the brain with high spatial and temporal resolution. Integrated biochemical sensing devices based upon flexible microelectrode arrays (MEA) have emerged as a powerful tool for such purposes; however, MEAs suffer from undesired biofouling and inflammatory reactions, while those with immobilized biologic sensing elements experience additional failures due to biomolecule degradation. Aptasensors are powerful tools for building highly selective sensors for analytes that have been difficult to detect. In this work, DNA aptamer-based electrochemical cocaine sensors were integrated on flexible MEAs and protected with an antifouling zwitterionic poly (sulfobetaine methacrylate) (PSB) coating, in order to prevent sensors from biofouling and degradation by the host tissue. In vitro experiments showed that without the PSB coating, both adsorption of plasma protein albumin and exposure to DNase-1 enzyme have detrimental effects on sensor performance, decreasing signal amplitude and the sensitivity of the sensors. Albumin adsorption caused a 44.4% sensitivity loss, and DNase-1 exposure for 24 hr resulted in a 57.2% sensitivity reduction. The PSB coating successfully protected sensors from albumin fouling and DNase-1 enzyme digestion. In vivo tests showed that the PSB coated MEA aptasensors can detect repeated cocaine infusions in the brain for 3 hrs after implantation without sensitivity degradation. Additionally, the same MEAs can record electrophysiological signals at different tissue depths simultaneously. This novel flexible MEA with integrated cocaine sensors can serve as a valuable tool for understanding the mechanisms of cocaine addiction, while the PSB coating technology can be generalized to improve all implantable devices suffering from biofouling and inflammatory host responses.

## 1. Introduction

In 2020, the Substance Abuse and Mental Health Services Administration reported that the number of people aged 12 years and older using illicit drugs reached 59.3 million, and of this population, 5.2 million (1.9%) used cocaine [1]. Among this staggering number of users, 1.4 million (4.3%) people are young adults aged 18 to 25, and 84,000 (0.3%) are adolescents aged 12 to 17 [1,2]. Cocaine is an extremely addictive psychostimulant that exhibits highly variable brain region specific effects [2,3,4,5,6,7,8,9]. It produces psychoactive and addictive effects primarily by affecting the brain’s limbic system, a set of interconnected regions that regulate pleasure and motivation, through competitive inhibition of the dopamine transporter [10,11,12,13,14,15].

A particularly interesting and devastating observation is that adolescents are more vulnerable to cocaine addiction [16,17]. Numerous environmental factors can contribute to this vulnerability, but substantial electrophysiological and behavioral evidence in rodents revealed that this vulnerability is biological in origin [16,17,18]. Previous studies have attempted to quantify how age affects cocaine concentration in the brain following acute cocaine administrations [16,18,19,20], but results are variable and inconsistent. The discrepancy is possibly due to limitations in current available analytic techniques. The gold standard technique for characterizing brain biochemical concentrations is microdialysis that exhibits low temporal (minutes to hours sampling period) and spatial (averaging over 0.0314 mm^3^ volume of tissue) resolution [18,21,22]. The large footprint of microdialysis probes also induce local tissue damage that significantly decreases extraction efficiency and causes under estimation of analyte concentrations [23,24,25,26,27]. Postmortem digested tissue analysis is another standard technique to investigate brain cocaine concentrations at various time-points following cocaine administration in the animal. This technique can detect cocaine and a wide range of relevant parameters (cocaine metabolites, receptors, cell types) with high selectivity and specificity, but it has extremely poor temporal resolution (single time point per animal) [28,29]. In the past 40 years since the discovery of the “cocaine high”, scientists have learned a great deal in the underlying mechanisms of cocaine’s physiological effects, but there remain many open questions on the mechanisms of cocaine addiction due to the lack of analytic techniques [3]. In order to fully understand the mechanism of action and develop effective therapies for cocaine addiction, a reliable tool is needed to measure *real-time* in vivo cocaine dynamics with high spatial and temporal resolution.

Microelectrode arrays (MEA) are powerful tools for brain electrophysiology recording and they are also a versatile platform for the incorporation of different functionalities to achieve multimodality [30,31,32,33]. One of the more useful modalities is biochemical sensing; there have been a few successful demonstrations of utilizing MEAs for in vivo biochemical sensing [34,35,36], but in vivo biochemical sensing with MEA devices remains a challenging task. Electrochemical aptamer-based sensors have emerged as a promising candidate for biomolecule sensing [37]. Aptamers are DNA or RNA strands that are artificially synthesized and selected to have high binding affinity to target molecules [38]. Affinity binding of target molecules to aptamers will result in reversible conformational changes of the aptamers and entrapment of the molecule within the aptamers’ chain. The reversible affinity binding of targets ensures the target molecule is not consumed or absorbed in the process [39]. For electrochemical aptasensors, aptamers are either used in combination with field effect transistors (FET) for sensing through surface charge change [40,41] or functionalized with electrochemical reporter molecules, such as methylene blue (MB), ferrocene, or anthraquinone to enable direct detection of analytes binding through electrochemical techniques [42,43]. Because of aptamers’ flexibility in target selection, a broad range of aptamers have been developed for different target molecules, ranging from small biomolecules such as cocaine, ATP, doxorubicin, tobramycin, dopamine, and serotonin [37,40,44,45,46,47,48,49,50,51] to proteins such as thrombin, PDGF, immunoglobulin G, immunoglobulin E, myoglobin, MUC1, and gene 32 protein [52,53,54,55,56,57,58].

An electrochemical aptamer-based cocaine sensor has been developed by Plaxco et. al., which involves immobilization of MB functionalized aptamer on a gold electrode surface and the use of square wave voltammetry (SWV) to detect an MB reduction current change that is proportional to the cocaine concentration. This aptasensor design has demonstrated excellent sensitivity and specificity in vitro [37,44,45]. By integrating cocaine aptamers onto implantable silicon MEAs, we showed successful in vivo cocaine sensing and electrophysiology recording from rat brains [35]. However, the sensor exhibited signal degradation 1 h after implantation [35]. Histology investigation revealed a layer of biological material composed of plasma proteins and microglia cells on the electrode sites that could compromise sensor performance [35,59]. The brain tissue foreign body response to the implanted silicon MEA devices has been well characterized by previous research and determined to cause recording quality decline [33,59,60,61,62,63,64]. A rich body of research has also shown that neural recording MEAs made from flexible polymer substrate cause less damage and inflammatory brain tissue response than their stiff counterparts [64,65,66]. In addition to the mechanical factors, surface chemistry is critical in the initial interaction between device and host tissue. Zwitterionic polymers carry positive and negative charges at a 1:1 ratio in each repeating unit. The high concentration of ions along the backbone gives zwitterionic polymers exceptional hydration power. Surfaces with zwitterionic polymer coatings are super-hydrophilic. Water molecules bind strongly to the zwitterionic surface and form a hydration layer that can inhibit protein adsorption and cell attachment [67,68,69,70,71,72]. Our previous studies have demonstrated that zwitterionic polymers can prevent non-specific protein adsorption and cell adhesion in vitro and are also beneficial in reducing glia cell adhesion and tissue response in vivo [72,73]. Another potential cause of sensor signal degradation could be aptamer degradation by extracellular DNase-1. In addition to the excellent anti-fouling potential, the zwitterionic polymer coating may also act as a physical barrier that blocks large biomolecules such as DNase-1 from interacting with the electrode surface and aptamers.

So far, no work has been done in using flexible MEAs to construct aptasensors for in vivo applications. To reduce undesired brain tissue response and protect implanted sensors from biofouling and DNase-1 in the extracellular space, zwitterionic polymer non-fouling coatings were applied as a protective coating on flexible MEA devices. We hypothesize that the use of flexible MEAs with zwitterionic coatings may synergistically improve aptasensors’ in vivo performance. In current work, we have first fabricated flexible MEAs using SU-8 substrate and integrated cocaine aptamer on the MEA sites. Then, zwitterionic poly (sulfobetaine methacrylate) (PSB) coating was applied to the flexible MEAs and the effects of the PSB coating and whether it helped maintain sensor stability was characterized in vitro and in vivo.

## 2. Materials and Methods

### 2.1. Materials

Copper(I) Bromide (CuBr 98%), imidazole (≥99%), bromoisobutyryl bromide (BIBB 98%), [2-(methacryloyloxy) ethyl]-dimethyl-(3-sulfopropyl) ammonium hydroxide (SBMA 97%), 2,2′-bipyridine (BPY, ≥99%), tetrahydrofluran (THF), tert-butyl chlorodimethylsilane (TBDMS, 97%), triethylamine (TEA), tetrabutylammonium fluoride (TBAF), and dopamine-HCl (99%) were purchased from Sigma-Aldrich (St. Louis, MO, USA).

Cocaine-targeting aptamer, 5′-HS-(CH_2_)_6_-AGACAAGGAAAATCCTTCAATGAAGTGGGTCG-(CH_2_)_7_-MB-3′ [35,37,45], was designed with a 5′ terminal thiol group (attached with a six-carbon linker) to facilitate direct covalent linkage to the gold surface and a 3′ terminal methylene blue (MB) group (attached with a seven-carbon linker) as an electrochemical reporter molecule. Aptamer was synthesized and dual HPLC purified prior to purchase from Integrated DNA Technologies, Inc. (Coralville, IA, USA). This specific cocaine-targeting aptamer has been well characterized to exhibit a 90 mM K_d_ [37]. All DI water and PBS (1×) used are DNase free.

### 2.2. MEA Fabrication for In Vitro and In Vivo Use

The surface MEA chip and flexible MEAs were fabricated following previous procedures with small modifications [34]. A Si wafer with a 1µm thick SiO_2_ layer (University Wafer Inc., South Boston, MA, USA) was first cleaned by sonicating in acetone, isopropanol, and DI water sequentially for 5 min each step. The wafer was then dried on a hot plate at 150 °C for 3 min, then the surface was cleaned and activated by O_2_ plasma using a reactive ion etcher (RIE, Trion Phantom III LT, Clearwater, FL, USA) for 120 s at 200 mTorr pressure and 150 Watts power. The wafer was then spin-coated with SU-8 2015 (MicroChemicals, Ulm, Germany) at 5000 rpm for 1 min and soft baked at 65 °C for 3 min and 95 °C for 5 min to evaporate solvent, then the wafer was exposed using a maskless aligner (MLA, MLA100, Heidelberg Instruments, Heidelberg, Germany) with a dose of 400 mJ/cm^2^. After exposure, the SU-8 first layer was post-baked at 65 °C for 3 min and 95 °C for 5 min, developed using SU-8 developer (MicroChemicals, Germany) for 1 min, and cleaned by isopropanol and DI water, Then, the wafer was hard baked at 200 °C, 180 °C, and 150 °C for 5 min each for SU-8 curing and allowed to cool down below 95 °C (step skipped for in vitro MEA fabrication). The wafer was then treated with O2 plasma to clean, activate, and roughen the SU-8 with RIE for 75 s at a pressure of 200 mTorr and 150 W power. The treated wafer was then spin-coated with AZ P4620 photoresist (MicroChemicals, Germany) at 5000 rpm for 1 min and baked at 105 °C for 5 min for resist curing. After baking, the wafer was exposed using MLA with a dose of 700 mJ/cm^2^, then developed using AZ400k 1:4 developer (MicroChemicals, Germany), cleaned by water rinse, and dried with N2 gas flow. A mild 120 s RIE O2 plasma treatment at pressure 600 mTorr and 60 W power was performed to clean the SU-8 before metal deposition. A 10 nm Ti adhesion layer and 100 nm Au layer were evaporated on the wafer using an Electron Beam Evaporator Plassys MEB550S (Marolles-en-Hurepoix, France). The metal was then lifted off in acetone overnight. The next day, the wafer was first rinsed with water, dried under N_2_ flow, and cleaned by O2 plasma for 60 s at 600 mTorr and 60 W, then spin-coated with SU-8 2015 for insulation layer at 5000 rpm for 1 min and soft baked at 65 °C for 2 min and 95 °C for 5 min. The wafer was then exposed using MLA with a dose of 400 mJ/cm^2^, post-backed, and developed with an SU-8 developer. The wafer was then cleaned with isopropanol and water, and was hard baked at 200 °C, 180 °C, and 150 °C for 5 min each to fully cure SU-8 and allowed to cool down below 95 °C. For in vitro use, the wafer was then cut into rectangular pieces (1.5 × 2 cm) containing an MEA and used for in vitro experiments. For in vivo use, the flexible MEAs were released from the wafer using buffered oxide etchant (1:7) (MicroChemicals, Germany) in an acid hood for 8 h to etch away the SiO_2_ layer. Customized PCBs were mounted with ZIF and Omnetics connectors. ZIF connectors were used to interface with flexible MEAs, and Omnetics connectors were used to interface with the potentiostat and electrophysiology recording system.

### 2.3. Aptamer Functionalization of Microelectrode Sites

**Scheme 1:**
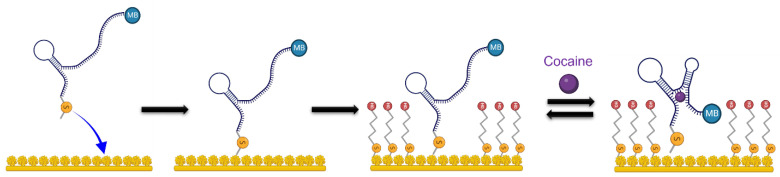
Schematic demonstration of the standard self-assembly process for aptasensors and their detection mechanism. Fuzzy Au was first deposited on electrode sites. Aptamers were then immobilized on the fuzzy Au surface after which an additional self-assemble monolayer of 6-mercapto-1-hexanol (MCH) was immobilized as the passivation layer. Upon binding of the cocaine, the conformation change of aptamer brings the MB tag closer to the surface, resulting in higher current response.

MEA electrode sites were coated with fuzzy Au using repeated multi-step chronoamperometry (−400 mV for 1 s followed by 0 V for 1 s, 240 cycles) in a solution containing 3.5 M HAuCl_4_ and 0.1 M NaCl in 1.5 wt% HCl, to increase the available gold surface area. Fuzzy gold coated electrodes were immediately functionalized with cocaine-targeting aptamer using well-established protocols [37]. Next, 5 mL aliquots of 100 mM aptamer solution were incubated in 15 mL of 50 mM tris-(2-carboxyethyl) phosphine hydrochloride (TCEP, prepared in PBS) for 1 h to reduce the 5′ thiol group for gold attachment. The reduced aptamer solution was then diluted to 5 mM with PBS for electrode functionalization. Fuzzy gold coated MEAs were functionalized by soaking in aptamer solution overnight. Finally, functionalized electrodes were soaked in 30 mM 6-mercaptohexanol (MCH, prepared in PBS) or hexanethiol (HT, prepared in PBS) for 2 h to block any unreacted gold surface. The resulting cocaine sensing probes were rinsed and stored in PBS solution in the dark at 4 °C until use (Scheme 1).

### 2.4. PSB Synthesis and Coating Procedure

PSB was synthesized following previous literature [72]. In brief, Dopamine-HCl (2.72 g, 14.3 mmol) and imidazole (4.3 g, 71 mmol) were dissolved in dry THF (50 mL) and added into a round bottom flask under N2 purge. Then, TBDMS (6.48 g, 43 mmol) was dissolved in dry THF and slowly added into the flask. The reaction was carried out at room temperature for 3 h. After filtration of the precipitate and removal of solvent, the final mixture was purified by silica gel chromatography with dichloromethane-methanol (4:1). 1H NMR (400Hz, CDCl3), δ[ppm]: 6.71–6.63 (m, 3 H), 3.49–3.44 (q, J = 8 Hz, 2 H), 2.73–2.70 (t, J = 8 Hz, 2 H), 1.90 (s, 2 H), 0.98–0.97 (d, J = 4 Hz, 18 H), 0.19–0.18 (d, J = 4 Hz, 12 H). 13C, NMR, δ[ppm]: 147.17, 146.25, 129.52, 121.82, 121.74, 121.52, 63.28, 41.71, 34.77, 32.73, 25.86, −3.88.

The resulting product (6 g, 14.3 mmol) was then dissolved in dry THF and transferred into a flask under N2 purge. Then, TEA (2.2 mL, 15.8 mmol) was mixed with 20 mL of THF and added into the flask. The mixture was cooled to 0 °C using an ice bath, then BIBB (3.95 g, 17.16 mmol) was mixed with 15 mL of THF and added into the flask dropwise with a dropping funnel. The reaction was carried out at room temperature overnight. After filtration of precipitate and removal of solvent, crude product was dissolved in ether, washed with NaHCO_3_ solution 3 times, dried with MgSO4, and passed through a silica gel chromatography with hexane-ethyl acetate (2:1). Pure product was collected after filtration and vacuum dried as white crystal. 1H NMR (400 Hz, CDCl3), δ[ppm]: 6.71–6.63 (m, 3 H), 3.49–3.44 (q, J = 8 Hz, 2 H), 2.73–2.70 (t, J = 8 Hz, 2 H), 1.90 (s, 2 H), 0.98–0.97 (d, J = 4 Hz, 18 H), 0.19–0.18 (d, J = 4 Hz, 12 H). 13C, NMR, δ[ppm]: 147.17, 146.25, 129.52, 121.82, 121.74, 121.52, 63.28, 41.71, 34.77, 32.73, 25.86, −3.88.

**Scheme 2:**
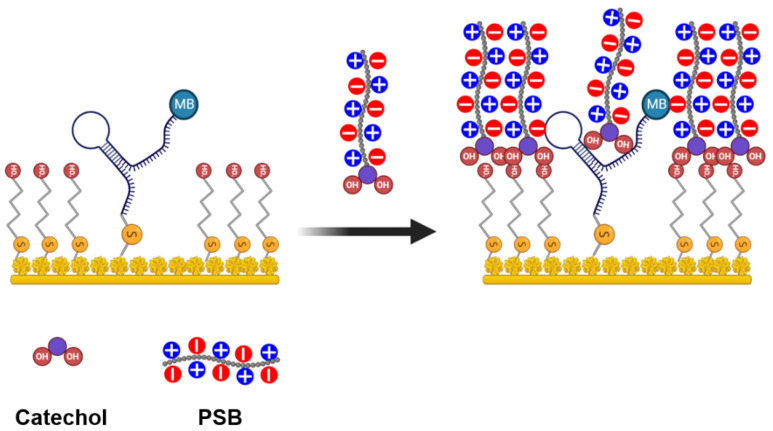
Schematic demonstration of PSB deposition on standard cocaine aptasensors. The fabricated standard cocaine aptasensors were immersed in 10 mM Tris buffer containing 2 mg/mL of PSB polymer for 2 h for coating deposition. The PSB polymers were anchored to the hydroxyl groups on the sensor surface through their catechol functional groups [74].

The molecular weight (MW) of PSB was measured to be 2.7 × 10^5^ g/mol using Agilent 1000 Series with a SEC column (YMC-Pack DL06S05-3006WT. 5 µm particle size, 60 Å pore size, 300 × 6.0 mm L × ID). To coat sensors with PSB, the substrates were immersed in 10 mM Tris buffer containing PSB (2 mg/mL) for 2 h and then rinsed with PBS. Fourier-transform infrared spectroscopy (FTIR) was performed using Nicolet^TM^iS20 (Thermo Fisher Scientific, Waltham, MA, USA). NMR spectra were measured on a Bruker ultra-shield 400 plus (Billerica, MA, USA) (Scheme 2).

### 2.5. Electrochemical Methods

All electrochemical procedures were conducted using a three-electrode design (working electrode: individual MEA electrodes; reference electrode: Ag/AgCl; counter electrode: platinum wire (in vitro experiments) or stainless-steel bone screw (in vivo experiments)). An Autolab potentiostat/galvanostat, PGSTAT128N (Metrohm, Herisau, Switzerland) was used for all square wave voltammetry (SWV) procedures and electrochemical Impedance spectroscopy (EIS) measurements (in vivo and in vitro). SWV potential was swept from −150 mV to −550 mV at 100 Hz with −5 mV step height and 25 mV pulse height.

### 2.6. In Vitro Experiment

**Figure 1:**
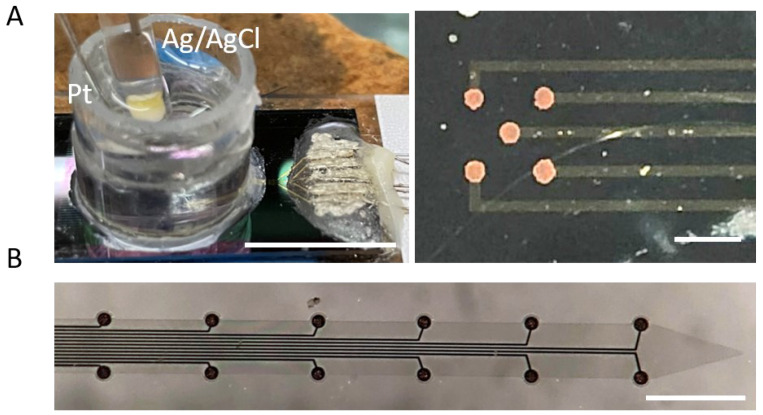
Optical images of the in vitro and in vivo MEAs. (**A**) In vitro silicon MEA chip assembly. Left: A 1 mL cloning ring was glued on the silicon MEA chip enclosing the microelectrode sites. Stainless-steel wires and silver paste were used for connecting the contact pads of the MEA chip to the potentiostat. The electrochemical cell was set up with a Pt counter electrode and Ag/AgCl reference, scale bar = 0.5 cm. Right: the 5 microelectrode sites are distributed in the 4 corners and the center of a 300 × 300 µm square. Scale bar = 300 µm. (**B**) Flexible SU-8 thin film MEA for in vivo experiments. 12 Au sites (35 µm diameter) with a spacing of 300 µm are distributed along both edges of the shank. Scale bar 300 µm.

Aptamer functionalized MEA chips were used as a robust in vitro testing platform. The set-up is shown in Figure 1 where stainless-steel wires (A-M systems, Sequim, WA, USA) were connected to individual contact pads through silver paste (Electron Microscopy Sciences, Hatfield, PA, USA) and insulated with rapid curing epoxy Devcon 14270 (ITW performance Polymers, Danvers, MA, USA) and fixed in place with UV curing dental cement (Prime Dental, Mount Pleasant, PA, USA). A 1 mL cloning ring was glued above the area containing the electrode sites to create a vial to hold liquid for in vitro electrochemical experiments (Figure 1A).

For the cocaine spiking assay, stock cocaine solutions of 250 mM, 50 mM, 5 mM, and 0.5 mM were first made. Then, 1 mL of PBS was added in the vial mount on MEA chip. Calibration was done by adding 0.5 mM, 5 mM, 50 mM, and 250 mM stock solutions into the 1 mL PBS stepwise. SWV measurements were taken after each stock solution addition.

For the protein adsorption assay, 1 mL of PBS and 10 mM cocaine solution was used to acquire blank and cocaine spiked responses, respectively. Measurements were taken before and after 1 h bovine serum albumin (2 mg/mL in PBS) exposure on PSB coated and non-coated sensors. The sensors were rinsed with PBS three times before taking measurements.

In the DNase assay, non-coated and PSB coated sensors were exposed to DNase-1 (1 U/µL) for 24 h at 37 °C [75]. SWV measurements were taken before and after exposure to DNase-1 for both blank and 10 mM cocaine solution. Sensors exposed to DNase-1 were rinsed three times with PBS before taking measurements.

The in vitro PBS injection experiment was performed in a 10 mL beaker. To make 0.6% agarose gel, 60 mg of agarose was first mixed with 10 mL of PBS in the beaker and microwaved for 20 s, then the beaker was cooled at 4 °C for 20 min to allow complete gelification. The MEA shanks were temporarily glued to a fused silica capillary (75 mm i.d., 180 mm o.d., Polymicro Technologies, Phoenix, AZ, USA) with 30% PEG. The capillary inlet was connected to a syringe using a lure lock PEEK™ female fitting (LabSmith, Inc., Livermore, CA, USA). PBS was delivered through the capillary using a Pump 11 Elite syringe pump (Harvard Apparatus, Holliston, MA, USA) in a Hamilton gastight 1705 syringe (Hamilton Co., Reno, NV, USA). The MEA shank was inserted into a 0.6% agarose gel 5 mm deep and 1 µL PBS was delivered at 6 different time points (20 s, 30 s, 40 s, 60 s, 90 s, 130 s). Additionally, 250 mM cocaine was delivered at 170 s using a pipette.

### 2.7. In Vivo Experiment

All animal procedures were approved by the University of Pittsburgh Institutional Animal Care and Use Committee. For all in vivo experiments, male Sprague Dawley (SD) rats (250–359 g: Charles River, Ashland, OH, USA) were anesthetized with isoflurane (2% by volume) and were immobilized in a stereotaxic frame. Body temperature was maintained at 37 °C using a Deltaphase isothermal pad (Braintree Scientific Inc., Braintree, MA, USA). Portions of the skull and dura were removed directly above the dorsal striatum (2.5 mm anterior to bregma, 2.5 mm lateral from bregma) to allow insertion of the flexible MEA into the dorsal striatum (5 mm below the cortical surface). Two other unmeasured positions in the contralateral hemisphere were drilled open for the placement of a stainless-steel counter electrode bone screw and an Ag/AgCl reference electrode (connected via salt bridge).

For direct local cocaine infusion experiments, MEA shanks were temporarily glued to a fused silica capillary (75 mm i.d., 180 mm o.d., Polymicro Technologies, Phoenix, AZ, USA) with 30% PEG. The capillary was positioned on the back side of the probe shank, opposite the electrode site openings (0.5 mm from the MEA’s tip). A representative image of the MEA/capillary assembly and surgery set-up is shown in Figure 6A. The capillary inlet was connected to a syringe using a lure lock PEEK™ female fitting (LabSmith, Inc., Livermore, CA, USA). Cocaine was delivered through the capillary using a Pump 11 Elite syringe pump (Harvard Apparatus, Holliston, MA, USA) in a Hamilton gastight 1705 syringe (Hamilton Co., Reno, NV, USA). For each animal, the SWV waveform was applied immediately upon insertion of the cocaine sensor and was repeated every 2 s (the maximum frequency allowed by the potentiostat at the assigned SWV waveform parameters) until the predetermined experimental endpoint. Baseline in vivo electrode drift was monitored for 30 min. At this point, cocaine was administered to all animals. For repeated direct local cocaine infusion experiments, one 1 µL bolus of 250 mM cocaine solution was first delivered to ensure the capillary was not blocked by PEG before implantation and then repeats of a 1 µL bolus of 250 mM cocaine solution was directly infused into the brain after implantation every 30 min for 3.5 h (for a total of 7 injections).

After the direct infusion experiments, the PCB was connected to a TDT 16 channel Omnetics head-stage. The neural signal was amplified with a 16 channel Medusa preamplifier and recorded with an RX5 processor at 25 kHz sampling rate (Tucker-Davis Technologies, Alachua, FL, USA). Neural signals were subsequently processed with custom MATLAB scripts and Offline Sorter (Plexon Inc., Dallas, TX, USA). Raw data was filtered between 300 Hz to 10 k Hz. Individual units were identified by using a fixed negative threshold value of 3.5 standard deviation. A 3D PCA feature space was used for sorting using the K-means clustering method. The range of units for K-means and Standard E m was chosen to be 2 to 5.

### 2.8. Data Analysis

Each SWV response was first filtered using a zero-phase, forward and reverse (using the filtfilt function on MATLAB), low-pass, third-order Butterworth digital filter with the 3 dB cutoff at a normalized frequency of 0.2 (2 Hz). The fit for the linear baseline was determined using a two-step peak extraction method consisting of an iterative peak localization algorithm. First, a linear baseline was initialized with two signal points on either side of a user-selected peak maximum voltage (−0.3 V). Signal points used to construct the baseline were iteratively updated to produce a final baseline which maximized the subtracted peak amplitude. The resulting linear fit intersected boundary points at either side of the MB peak profile. The five data points immediately adjacent to the upper and lower bounds were then modeled using linear fitting and subtracted from the raw SWV response for the purpose of peak extraction as demonstrated in Figure S1.

For in vitro experiments, the percent current response was calculated by subtracting the blank current amplitude from the 10 mM response, and this difference was divided by the blank response.

For in vivo experiments, the peak current over time was first divided into six 30-min segments, and each segment was first zeroed by subtracting its minimum value to correct for background drifting. Data points between 15–20 min were averaged and used as the baseline response for each channel. Every data point was then subtracted by this baseline and the calculated difference was divided by the average baseline to acquire a normalized percent current response in vivo.

Statistical analysis and results plotting was done using GraphPad Prism 9.5.0 and MATLAB 2019a. One-way ANOVA with Dunnett’s post hoc test and Welch’s *T* test methods were used. Data described in text are mean ± SD unless specified otherwise.

Schemes were drawn with BioRender.

## 3. Results 

Figure 1 shows the final product of a microfabricated in vitro silicon MEA chip assembly and an in vivo flexible MEA. The in vitro silicon MEA chips had five Au sites distributed at the four corners and the center of a 300 × 300 µm square on the silicon chip insulated by 3 µm thick SU-8 insulation (Figure 1A). The in vivo flexible MEAs were fabricated using SU-8 as both the substrate and insulation material. The total length of the shank was 5 mm. The shank had a width of 150 µm and thickness of 15 µm. There were twelve Au sites (35 µm diameter) distributed along both edges of the shank. The vertical spacing between sites was 300 µm (Figure 1B). The averaged impedance of the MEAs as fabricated, after fuzzy Au deposition, and after aptamer/MCH immobilization is shown in Figure S2. Only small variations were observed among the 19 samples measured, indicating that the fabrication process is reproducible and robust (Figure S2).

**Figure 2:**
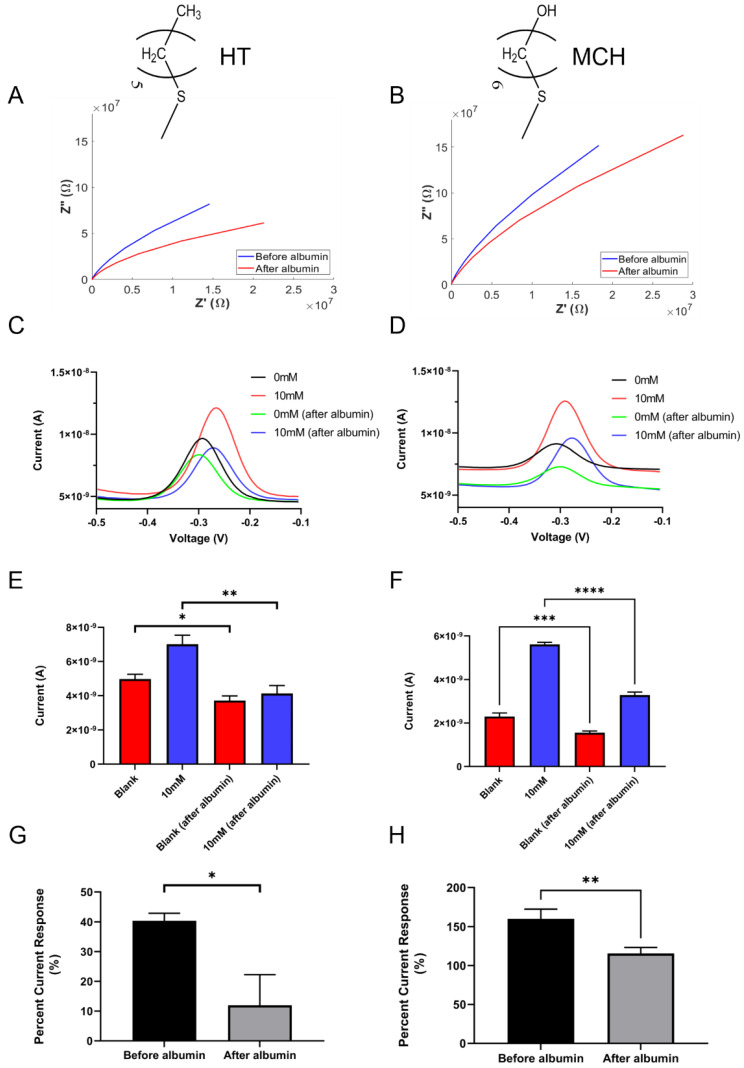
In vitro protein fouling assay for testing effects of different passivation layers. Hydrophobic hexanethiol (HT) is compared with standard hydrophilic 6-mercapto-1-hexanol (MCH). (**A**) Nyquist plot before and after albumin exposure for the HT group. The aptasensor became more resistive after exposed to albumin. (**B**) Nyquist plot before and after albumin exposure for the MCH group. The aptasensor also became more resistive after being exposed to albumin. (**C**) SWV waveform of the HT group before and after albumin exposure. A clear peak amplitude drop can be observed. (**D**) SWV waveform of the MCH group before and after albumin exposure. A background current drop can be observed. (**E**) Quantification of peak current for the HT group. Peak current for both blank PBS and 10 mM cocaine showed significant decrease in amplitude after albumin exposure. One-way ANOVA, n = 5. (**F**) Quantification of peak current for the MCH group. Peak current for both blank PBS and 10 mM cocaine also showed a significant decrease in amplitude after albumin exposure. One-way ANOVA, n = 19. (**G**) Percent current response to 10 mM cocaine for the HT group after albumin exposure. Sensors showed significantly decreased signal towards cocaine after protein exposure. Welch *t* test, n = 5. (**H**) Percent current response to 10 mM cocaine for the MCH group after albumin exposure. Sensors also showed significantly decreased signal towards cocaine after protein exposure. Welch *t* test, n = 19. * *p* < 0.05, ** *p* < 0.01, *** *p* < 0.0005, **** *p* < 0.0001. All data are represented by mean + SEM. (**A**–**D**) SEM removed for clarity.

First, we examined the effects of plasma protein fouling on sensor performance in vitro. The protein fouling test was carried out by soaking the aptasensor in 2 mg/mL albumin, the most abundant plasma protein [76], for 1 h. At first glance, both the hexanethiol (HT) and the 6-mercaptohexanol (MCH) groups showed increased resistivity after protein fouling based the Nyquist plots (Figure 2A,B).

Protein fouling had different effects on MCH and HT capped sensors based on the SWV waveform. The background current decreased for the MCH group but stayed stable for the HT group (Figure 2C,D). Additionally, the methylene blue reduction peak at ~ −0.3 V decreased for both the blank and the 10mM cocaine response on both groups after protein exposure (Figure 2C,D). The HT treated sensor after protein adsorption showed a similar response for blank and 10mM cocaine solution (Figure 2C).

The aptasensors’ peak current amplitudes in response to blank PBS and 10 mM cocaine solutions were extracted and compared across groups (Figure 2E,F). For the HT group, both the blank and the 10 mM current response amplitudes significantly decreased after albumin exposure, and current amplitudes of the blank and the 10 mM cocaine responses were very similar (Figure 2E), indicating a loss of sensitivity to cocaine-induced folding of the aptamer. The sensitivity of the HT-capped sensors to cocaine dropped significantly from 40.4 ± 5.7% to 12 ± 23% (Figure 2G). The MCH group also displayed a significant decrease in current amplitudes for both the blank and the 10 mM response after the sensors were exposed to albumin (Figure 2F). The MCH group also showed a 44 ± 15% sensitivity loss (Figure 2H), but still responded to a 10 mM cocaine solution (116 ± 33%). These results indicate that protein fouling can negatively affect aptasensors’ performance.

**Figure 3:**
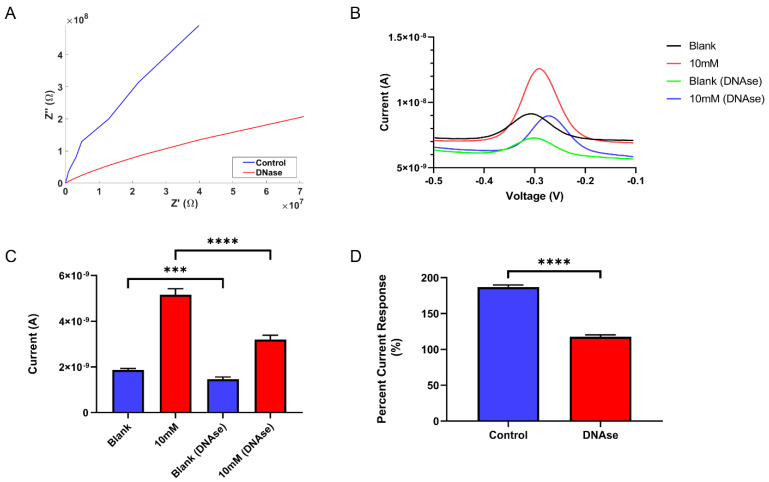
In vitro DNase exposure test. The aptasensors’ performance was compared before and after exposure to DNase-1. (**A**) Nyquist plot of sensors before and after DNase-1 exposure. Sensors became more resistive after DNase-1 exposure. (**B**) SWV waveform of sensors before and after DNase-1 exposure. A decrease in background current and MB reduction peak size can be seen. (**C**) Quantification of peak current. A significant amplitude decrease is observed for both blank and 10 mM cocaine response after exposure to DNase-1. One-way ANOVA, n = 8. (**D**) Percent current response characterization. The sensor’s sensitivity towards cocaine significantly decreased after being exposed to DNase-1. Welch’s *t* test, n = 8. *** *p* < 0.0005, **** *p* < 0.0001. all data mean + sem. (**A**,**B**) SEM removed for clarity.

Next, we examined the effects of DNase-1 on the aptasensors’ performance. Cocaine aptamers used in this work were double-stranded cDNA -a perfect target for DNase-1 enzyme. The cocaine sensing performance of the sensors was compared before and after exposure to DNase-1 for 24 h. After DNase-1 exposure, sensors became more resistive based on the Nyquist plot (Figure 3A). A reduction in peak size at ~−0.3 V and background current was observed for both the blank and the 10 mM cocaine solution response (Figure 3B). The peak current amplitude was then extracted and quantified, and results are summarized in Figure 3C. Both the blank and 10 mM responses showed a significant decrease in current amplitude after exposure to DNase-1 (Figure 3C). Additionally, the sensitivity of the sensor significantly decreased by 57.2 ± 7.3% after DNase-1 exposure (Figure 3D). 

**Figure 4:**
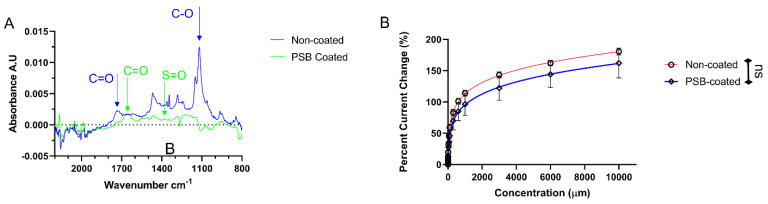
PSB coating characterization with FTIR and calibration curves. Pre-synthesized PSB polymers were dissolved in 10 mM Tris buffer and the aptamer functionalized MEAs were submerged in PSB solution for 2 h at room temperature. (**A**) FTIR spectrum of PSB coating. A Au coated 1 cm × 0.5 cm Si wafer was used for FTIR characterization of the coating. The blue arrow indicates characteristic peaks of C-O (1085–1050 cm^−1^) from MCH and C=O (1720–1706 cm^−1^) bond from aptamers. The green arrow indicates C=O (1680 cm^−1^), S=O (1372–1335 cm^−1^) from the PSB polymer. (**B**) The calibration curves of non-coated and PSB coated sensors in vitro. The coating did not affect sensors’ sensitivity. Mean ± SD, Modified Langmuir model, n = 6.

To protect the aptamer functionalized sensor surface from non-specific protein adsorption and possible DNase-1 insult in vivo, a zwitterionic PSB coating was utilized. PSB was synthesized via Atom Transfer Radical Polymerization (ATRP). The fabricated sensors were immersed in PSB polymer solution for 2 h for coating deposition. PSB polymers were anchored to the sensor surface through the catechol functional groups [72]. To characterize and confirm the coating chemistry with FTIR, Au plated Si wafers (1 cm × 0.5 cm) were functionalized with the same method as the MEAs and used as a substrate for PSB coating. FTIR spectra showed surface chemistry changes before and after PSB deposition. Before PSB coating, characteristic primary alcohol C-OH (1085–1050 cm^−1^) bonds from MCH and C=O (1720–1706 cm^−1^) bonds from aptamers were present. After PSB coating, secondary amide C=O (1680 cm^−1^) bonds and sulfonate S=O (1372–1335 cm^−1^) bonds from the PSB polymer were present (Figure 4A), indicating successful PSB attachment.

After confirming successful coating of PSB on the sensors, the sensitivity of PSB-coated sensors was compared with non-coated sensors. A step-wise cocaine spiking assay was performed to acquire and compare calibration curves for PSB coated and non-coated sensors. A clear peak current increase proportional to cocaine concentration can be seen in SWV waveforms of the calibrations (Figure S4). The addition of PSB coating did not alter the sensitivity of the sensors since the calibration curve of non-coated and PSB coated sensor are same (Figure 4B). Additionally, the impedance did not change after coating PSB onto the sensor (Figure S3).

**Figure 5:**
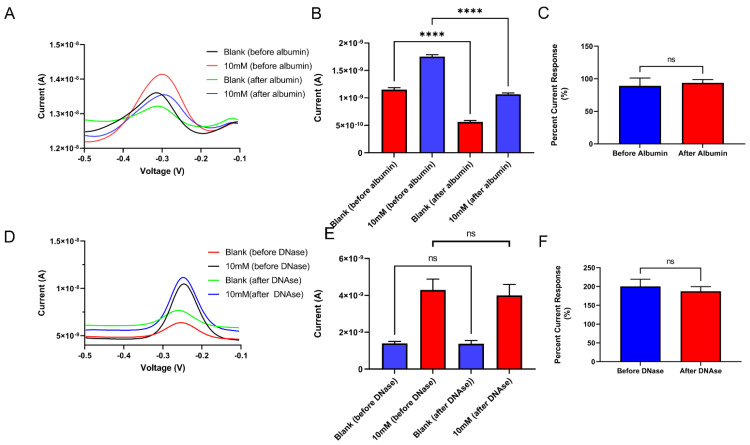
The effects of albumin adsorption and DNase-1 treatment on sensors with PSB coating in vitro. (**A**) SWV waveform of PSB coated sensors before and after albumin exposure. Fluctuations in background current and peak size changes were observed. (**B**) Quantified peak current comparison. Sensors showed significantly decreased current amplitude for both blank and 10 mM cocaine solution after exposure to albumin. One-way ANOVA, n = 25. (**C**) Percent current response quantification. PSB coated sensors showed no change in sensitivity towards cocaine after albumin exposure. Welch’s *t* test, n = 25. (**D**) SWV waveform of PSB coated sensors before and after DNase-1 exposure. An increase in background current was observed. (**E**,**F**) Quantified peak current and percent current response comparison. PSB coated sensors showed no signs of degradation after DNase exposure. Current amplitude (One-way ANOVA, n = 8) (**E**) and percent current response (Welch’s *t* test, n = 8) (**F**) were stable after exposure to DNase. **** *p* < 0.0001. all data mean + SEM. (**A**,**D**) SEM removed for visual clarity.

Next, the PSB coatings’ ability to resist protein fouling and protect the aptamer from DNase-1 was tested in vitro. PSB coated sensors were exposed to albumin solution (2 mg/mL) for 1 h. SWV showed fluctuations in background current and changes in peak size (Figure 5A). Quantified peak current amplitudes of PSB coated sensors significantly decreased for both blank and 10 mM cocaine solution (Figure 5B), but the sensor sensitivity was unaffected before and after albumin exposure (89 ± 69% before, 94 ± 25% after) (Figure 5C).

On the other hand, after exposure to DNase-1 for 24 h, SWV waveforms of PSB coated sensors showed elevated background current for both the blank and the 10 mM response after DNase-1 exposure (Figure 5D). The quantified peak current was also compared before and after DNase exposure (Figure 5E). No changes in current amplitude were observed for both the blank and the 10 mM cocaine responses (Figure 5E). The sensitivity towards cocaine (Figure 5F) was not affected after exposure to DNase-1 (200 ± 54% vs. 187 ± 35%).

**Figure 6:**
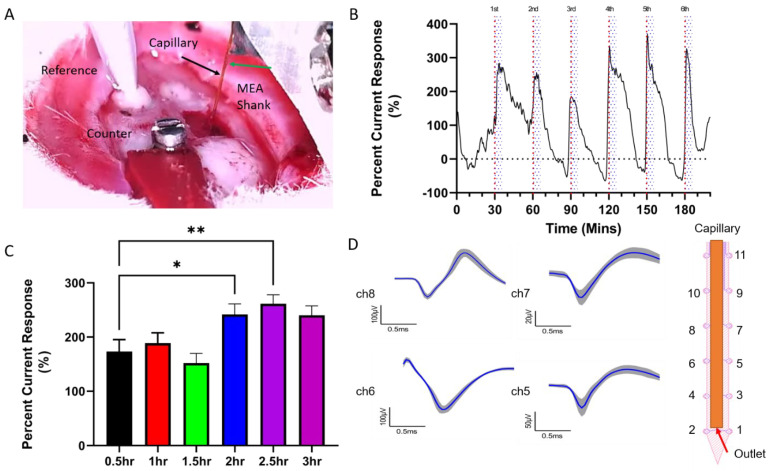
In vivo direct cocaine infusion surgery set-up, data analysis, and representative electrophysiology recordings of single unit waveforms. (**A**) A three-electrode set-up was used for in vivo cocaine sensing. Two holes were drilled on the contralateral side for placement of an Ag/AgCl reference electrode and a stainless-steel screw counter electrode. The flexible MEA shank was implanted 5 mm deep in the striatum using the capillary as shuttle. (**B**) Average percent current response to repeated cocaine injection over 200 min across 3 MEAs implanted in 3 rats. The red dashed line indicates the timing of repeated cocaine infusion every 30 min. The blue shaded regions indicate the 5 min time bins used to average the current response at different time points. Total number of electrode sites n = 9. (**C**) Average percent current response every 0.5 h from 3 implanted MEAs in 3 rats. At the 2 h and the 2.5 h time point, a significant increase in response was observed. Total number of electrode sites n = 9, * *p* < 0.05, ** *p* < 0.01. mean + SEM. One-way ANOVA, Dunnett’s test, 0.5 h response set as control. (**D**) Scheme of electrode site mapping, location of the capillary outlet, and representative single unit waveforms from electrophysiology recordings. The probe shank is 150 µm wide and vertical spacing of electrode sites is 300 µm. Capillary outlet is aligned with channel 1 and channel 2. channel 5 and 6 and | channel 7 and 8 are groups of electrodes at the same depth but recorded different units that have distinct waveforms.

Flexible MEAs functionalized with aptamers and coated with PSB were implanted into the striatum of the rat brain and coupled with a direct cocaine infusion strategy to evaluate the sensors’ stability in vivo. The in vivo experiment used a 3-electrode setup as demonstrated in Figure 6A. Two holes were drilled on the contralateral side of the cranial window for placement of the Ag/AgCl reference electrode and stainless-steel screw counter electrode. A custom 3D printed probe holder was used to mount the PCB interfacing with the flexible MEA (Figure 6A). The flexible MEA shank was attached to a fused silica capillary using polyethylene glycol (PEG), which served the dual purposes of insertion shuttle and injection channel. The assembly was manually inserted into the striatum of the rat brain using a surgical stereotaxic device (Figure 6A).

A 1 µL cocaine infusion (250 mM) was delivered via the capillary every 30 min for up to 3 h. The SWV was applied continuously as soon as the MEA was implanted into the striatum. The normalized response level was plotted over a 3.5 h time window (Figure 6B). The red dashed lines indicate timing of 6 repeated cocaine infusions and the blue shaded regions indicate the 5-min time bin used for quantifying the average percent current response at different time windows (Figure 6B). The current response levels at different time points were compared to the first 30 min time point and a significant increase in response level was observed at 2 h and 2.5 h time points comparing to 0.5 h (Figure 6C). Sensor performance was stable and no significant degradation in signals was observed. 

An in vitro PBS injection experiment was performed to rule out possible motion artifacts from the flux of solutions at the outlet. The sensors were assembled using the same method as in vivo and inserted into a 0.6% agarose gel. 1 µL PBS was delivered at different time points (20 s, 30 s, 40 s, 60 s, 90 s, 130 s), and 1 µL 250 mM cocaine solution was delivered at 170 s. No motion artifacts were observed after PBS injections and a clear response to cocaine was observed after the cocaine injection (Figure S5).

After the sensing experiment was finished, the Ag/AgCl reference electrode was removed from brain surface and the reference and counter wires from PCB were shorted and connected to the counter screw for electrophysiology recording experiments. The aptamer functionalized channels were also capable of recording high quality single units at different sites with high peak to peak amplitude (from ~50 µV to ~200 µV) (Figure 6D). The probe channel mapping and location of the capillary outlet is shown in Figure 6D. The vertical spacing of the electrode sites is 300 µm and lateral spacing is 150 µm, covering 1.2 mm of tissue depth. The outlet is aligned with channel 1&2 and only channels 1–4 were used for sensing experiments. Interestingly, channels at the same depth (channel6 vs. channel 5, channel 8 vs. channel 7) recorded different units. The stream data of each unit can be found in Supplemental Information (Figure S6).

## 4. Discussion

### 4.1. Effects of Non-Specific Protein Adsorption

Aptasensors require binding of target molecules to the aptamer and a conformational change to induce a detectable electrochemical signal change. Both mechanisms are crucial for signal generation. Adsorbed proteins could form a barrier layer (as shown in the EIS data) that prevents the aptamer from capturing the target molecules and/or undergoing the three-dimensional conformational change. This hypothesis is consistent with the observations from the albumin fouling assay (Figure 2), which clearly demonstrated that physical protein fouling can indeed negatively impact sensor performance.

The hydrophilic MCH passivation layer has been indicated in literature to have anti-fouling effects [73,74]. Therefore, we first investigated the effects of passivation layer hydrophobicity by comparing sensors’ performance after protein exposure between hydrophilic MCH and hydrophobic HT passivation layers. HT was used as a hydrophobic version of the passivation layer that has the same length of carbon chains as MCH. The HT group had a much lower percent current response compared to the MCH group before albumin exposure (40.38% vs. 159.9%). This could be attributed to the chain conformation and packing density difference of the self-assembled monolayer (SAM) used for the passivation layer. Previous research has shown that aptasensors’ signal is sensitive to the nature of the alkanethiol passivation layer (carbon chain length, hydrophobic vs. hydrophilic, layer integrity, etc.) [44,48].

After albumin exposure, sensors with the hydrophobic HT passivation layer were almost non-responsive to the 10 mM cocaine (Figure 2D), while sensors with hydrophilic MCH layer partially preserve the sensitivity. However, even with MCH, the sensor still suffered a significant sensitivity decline (44 ± 14%) after protein fouling (Figure 2H). Summarizing all the observations from the albumin fouling assay, these results clearly indicate that non-specific protein fouling could play a major role in decreasing sensors’ performance in vivo.

### 4.2. Effects of DNase-1 Enzyme Digestion

In vivo sensors are exposed to body fluids which contain a variety of enzymes and reactive species that can degrade the sensor. Specifically, aptamer-based sensors are vulnerable to DNases and RNases. DNase-1 is an enzyme that can effectively break down both single-stand and double-stand DNAs [75,77,78]. The cocaine aptamers used in this work were double-stranded cDNA, a perfect target for DNase-1. The peak current amplitude and percent current response drops of the sensors support the idea that aptamers were being destroyed by DNase-1 (Figure 3). A larger decrease in the percent current response was seen after DNase-1 exposure compared to protein adsorption (57 ± 7.3% vs. 44 ± 14%). It is also possible that besides DNase-1’s enzymatic digestion effects, fouling of DNase-1 can also play a role in reducing sensor performance. Both significantly decreased signal amplitude (peak current) and sensor sensitivity (percent current response), indicate that DNase-1 can indeed significantly degrade the sensors’ performance. This shows that DNase-1 could be one of the major factors affecting aptasensors’ in vivo performance, as endogenous DNase-1 exists both in the extracellular space and serum [75,77,78].

### 4.3. PSB Coating and Its Protective Effects In Vitro

Antifouling zwitterionic polymers have shown great potential in reducing the tissue foreign body response [58,70,71], and our previous works have demonstrated that PSB coating can prevent microglia cell adhesion and reduce inflammatory response, maintaining a healthy neural tissue-electrode device interface in vivo [72,73]. For this work, the pre-synthesized PSB containing mussel mimetic catechol adhesion end groups was chosen for facile surface immobilization. The coating deposition only involves a single immersion step to coat the MEA sensors. FTIR spectra clearly showed functional group changes after PSB coating. The alcohol C-OH peak from MCH disappeared while the C=O and S=O peaks from the PSB polymer appeared after coating. PSB polymer brushes can fill the spaces between aptamers, preventing protein interactions with the sensor surface and aptamers.

The immediate concern for this approach was whether the addition of the PSB coating would affect the aptamers’ folding capability. Thus, a calibration was done before and after PSB coating for benchmarking the sensors’ performance after PSB coating. The calibration curve was similar before and after coating, confirming that the PSB coating did not cause any unwanted effects on sensors’ sensitivity (Figure 4). The PSB coated sensors were further tested with an albumin adsorption assay and a DNase-1 assay in vitro. The PSB coated sensors maintained stable sensitivity after albumin exposure (Figure 5), but still experienced peak current amplitude loss. It is possible that the albumin was still able to interact with aptamers at the exposed top space and a longer polymer chain might help mitigate this effect. On the other hand, PSB coated sensors showed stable current amplitude and sensitivity after exposure to DNase-1. This demonstrated that PSB polymer brushes are very effective at stopping DNase from interacting with and digesting the aptamers. These results show that the PSB coating strategy is an effective method to protect aptasensors from non-specific protein adsorption and DNase-1 digestion in vitro while not affecting the sensor’s sensitivity.

The PSB coating method reported here, specifically the use of catechol functional groups for zwitterionic polymer PSB immobilization, has been reported in a few publications including our own (summarized in Table S1 in the Supplementary Information). The majority of these works were conducted to demonstrate the antifouling properties of the catechol PSB coating in general biomedical applications without testing in a specific target application [69,79]. One work used a catechol and zwitterion bifunctionalized PEG polymer, to protect an ATP aptasensor for ATP detection in biological media in vitro, but no in vivo testing has been done [80]. Our previous work used this catechol functionalized PSB polymer along with polydopamine to coat stiff Si-based MEAs and demonstrated a significant reduction in inflammatory tissue reaction by the PSB/PDA coating in vivo [72]. However, that study did not investigate the functional performance of the MEA. In the current work, we optimized the PSB coating specifically for cocaine aptasensors and investigated the protective effect of PSB against protein adsorption and DNase digestion, the latter of which has never been reported. Furthermore, to the best of our knowledge, this is the first in vivo demonstration of catechol functionalized PSB coating in an implantable MEA biosensor.

### 4.4. Stable In Vivo Sensing Performance and Electrophysiology Recording Capability with PSB Coating

To ensure a well-controlled and robust response in vivo, repeated and direct infusions of cocaine close to the MEA sensors were utilized to evaluate sensor performance over time. Since the aptamer and target binding is affinity based, as the tissue cocaine concentration goes down, the bound cocaine will dissociate from the aptamer. This and the continued tissue clearance of the cocaine enables the repeated measurements in order to evaluate the sensor’s stability over time. A capillary was used for fluid delivery, which also functioned as an insertion shuttle for implanting the flexible MEA devices. The overall percent current response level was stable except for the 2 h and 2.5 h time points, both of which showed significantly higher current responses compared to the initial time point (0.5 h). This could be attributed to cocaine retention in surrounding tissue as the infusion of cocaine was at a relatively high concentration and high rate compared to the clearance rate of cocaine in the brain reported by literature [22,81,82]. Compared with our previous work with stiff Si MEAs where the sensors showed decreasing current responses 1 h after implantation [35], the flexible MEA sensors showed a more stable detection overall without any signs of degradation.

The use of Au sites of similar size for electrophysiology recording is common in commercial MEAs and has been previously reported by us and others [32,35,65,83], but whether these electrodes can still record neural activity after the surface functionalization and PSB coating remains to be tested. We compared the electrical properties of the un-functionalized Au electrode sites and those that have been aptamers/MCH functionalized and PSB coated (Figures S2 and S3). We found the 1 kHz impedances of all the electrodes, with or without the biofunctionalization and PSB coating, fell within the range of being able to record single unit neural activity (<2 MΩ at 1 kHz) [84]. Indeed, when tested in vivo, the aptamer functionalized and PSB coated MEAs were able to record high quality single units from the striatum and the MEA design allowed sampling from different tissue depths with good spatial resolution (Figure 6D and Figure S6).

This work primarily focused on the effects of protein adsorption and DNase-1 on aptasensors’ in vivo sensing performance, however, other factors such as thermal stability or salt stability of the aptamers could also contribute to signal degradation. Previous research has demonstrated that adding phosphorylated nucleotides and altering nucleotides’ sequence can make aptamers more resilient to thermal and salt challenges [85,86]. Thus, this PSB coating strategy can be synergistically used with improved aptamer structure and sequence designs to combat the complex and harsh in vivo environment.

The acute experimental setup presented here to evaluate sensor performance could not fully utilize the potential of flexible MEA-based aptasensors, because the stiff capillary for cocaine delivery had to stay close to the MEA shank throughout the experiment. For future experiments, temporary shuttles with small footprints can be used to minimize insertion injury and can be removed to allow better tissue healing. This in turn could benefit tissue/device integration under chronic conditions. There have not been any successful attempts of using aptasensors for chronic in vivo applications. Although this work only studied protection effects of the PSB coating up to 3 h, no significant degradation in sensor performance was observed which is promising for future longer-term testing. Upon further characterization and optimization of in vivo stability, this integrated MEA aptasensor can be used to track real-time cocaine concentrations and record electrophysiology at high temporal resolution over time, making it a perfect tool for cocaine addiction studies.

## 5. Conclusions

In conclusion, we characterized the negative effects of protein adsorption on aptasensors’ background and signal current amplitude, as well as sensitivity. We determined in vitro that the hydrophilic MCH passivation layer better preserved the sensitivity of the aptasensor compared to a hydrophobic HT layer, but the aptasensor still suffered significant sensitivity loss. Additionally, we investigated the effects of DNase-1 on sensors’ performance and observed significant loss of both signal current amplitude and sensitivity in vitro. We utilized a zwitterionic PSB coating to protect the sensors from protein fouling and DNase-1 insult and demonstrated efficacy in vitro. We were able to implant flexible MEA aptasensors with PSB coatings and showed stable sensing performance in vivo for at least 3 h. The coating can be easily applied to a variety of device substrates and does not negatively impact the electrochemical sensing and neural recording capability. This integrated technology enables electrophysiology recording and cocaine sensing in the same brain region with high spatial and temporal resolution. It could serve as a highly valuable tool to help decipher cocaine addiction mechanisms. This technology platform can also be used to construct muti-sensors with a combination of different aptamers to detect multiple neurochemicals. The PSB coating method can be generalized to other biochemical sensing techniques for improving both acute and chronic in vivo sensor performance.

## Data Availability

The data presented in this study are available on request from the corresponding author.

## Supplementary Materials

The following supporting information can be downloaded at: https://www.mdpi.com/article/10.3390/mi14020323/s1, Figure S1: SWV data analysis demonstration; Figure S2: Impedance of sensors as fabricated, after fuzzy Au deposition, and after aptamer/MCH immobilization; Figure S3: Impedance of sensors before and after PSB coating; Figure S4: SWV waveform for calibrations comparison before (A) and after (B) PSB coatings; Figure S5: In vitro PBS injection experiment in 0.6% agarose; Figure S6: In vivo electrophysiology recording spike stream data; Table S1: Summary and comparison of previous works with PSB catechol zwitterionic polymer coatings.
