# Supplementary material for: Zwitterionic Polymer Coated and Aptamer Functionalized Flexible Micro-Electrode Arrays for In Vivo Cocaine Sensing and Electrophysiology
Source: Micromachines (Basel). 2023 Jan 27;14(2):323. doi: 10.3390/mi14020323 (PMC9967584; doi:10.3390/mi14020323)
Supplement: Supplementary file 1 [file micromachines-14-00323-s001.zip › micromachines-2135462-supplementary.pdf]

micromachines

MDPI

Supplementary Materials: Zwitterionic Polymer Coated and Aptamer Functionalized Flexible Micro-Electrode Arrays for In Vivo Cocaine Sensing and Electrophysiology

Bingchen Wu [1,2], Elisa Castagnola [3] and Xinyan Tracy Cui [1,2,4,*]

[1] Department of Bioengineering, University of Pittsburgh, Pittsburgh, PA 15213, USA
[2] Center for the Neural Basis of Cognition, Pittsburgh, PA 15213, USA
[3] Department of Biomedical Engineering, Louisiana Tech University, Ruston, LA 71272, USA
[4] McGowan Institute for Regenerative Medicine, Pittsburgh, PA 15219, USA
* Correspondence: xic11@pitt.edu

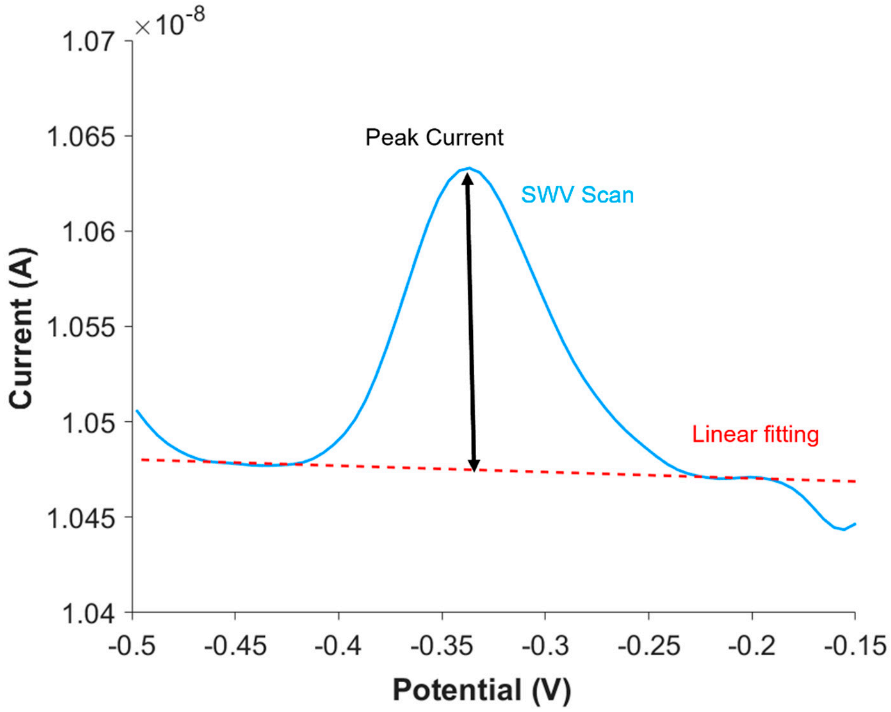

Figure S1. SWV data analysis demonstration. Using linear fitting method, a straight line is fitted as the baseline (Red dotted line). By subtracting this baseline from SWV scan (blue solid line), maximum peak current is extracted and used for peak current quantification (maximum distance of black double side arrow). .

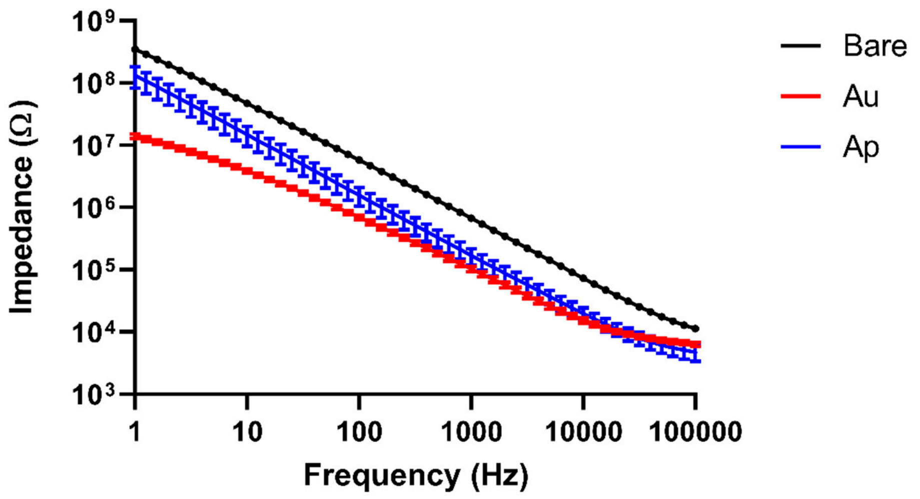

Figure S2. Impedance of sensors as fabricated, after fuzzy Au deposition, and after aptamer/MCH immobilization. The variation among different MEAs is small, indicating the fabrication process is robust and reliable. N =19.Mean±SEM.

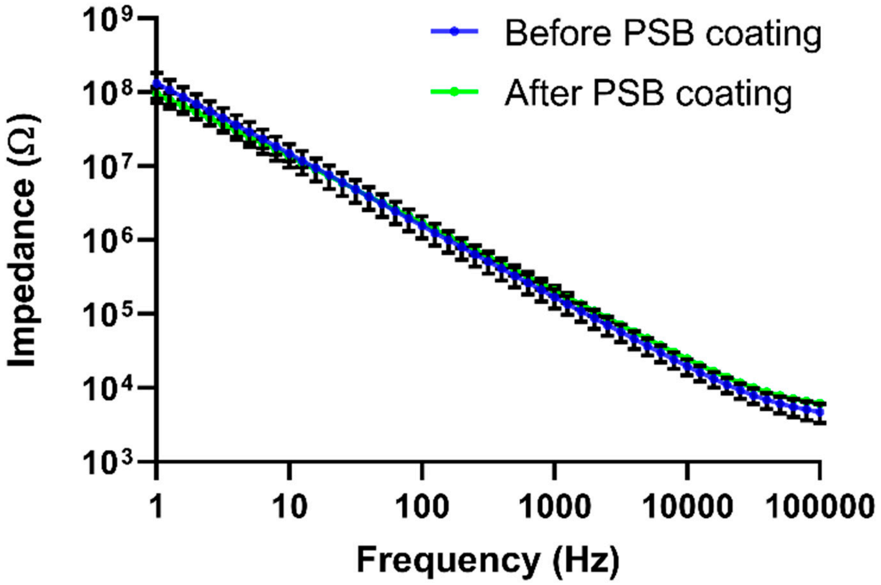

Figure S3. Impedance of sensors before and after PSB coating. No difference is observed before and after PSB coating was applied. N=8. Mean ± SEM.

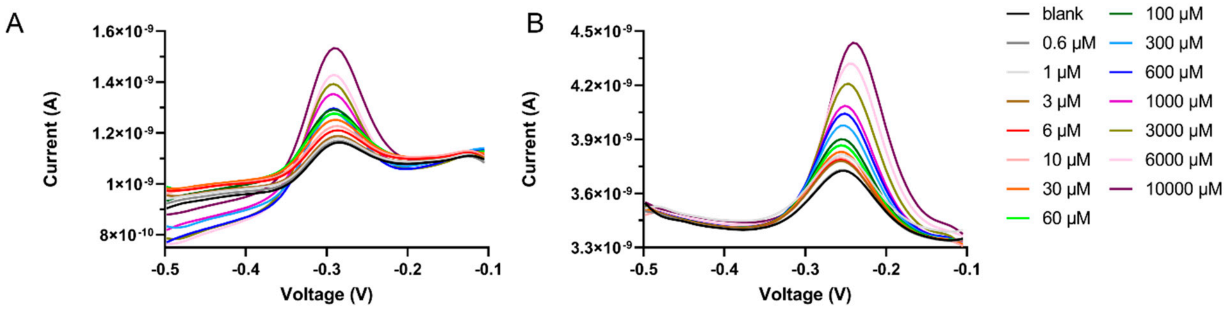

Figure S4. SWV waveform for calibrations comparison before (A) and after (B) PSB coatings. N=6. SEM removed for clarity. Clear peak current increase proportional to cocaine concentration can be seen. N=6, SEM removed for clarity.

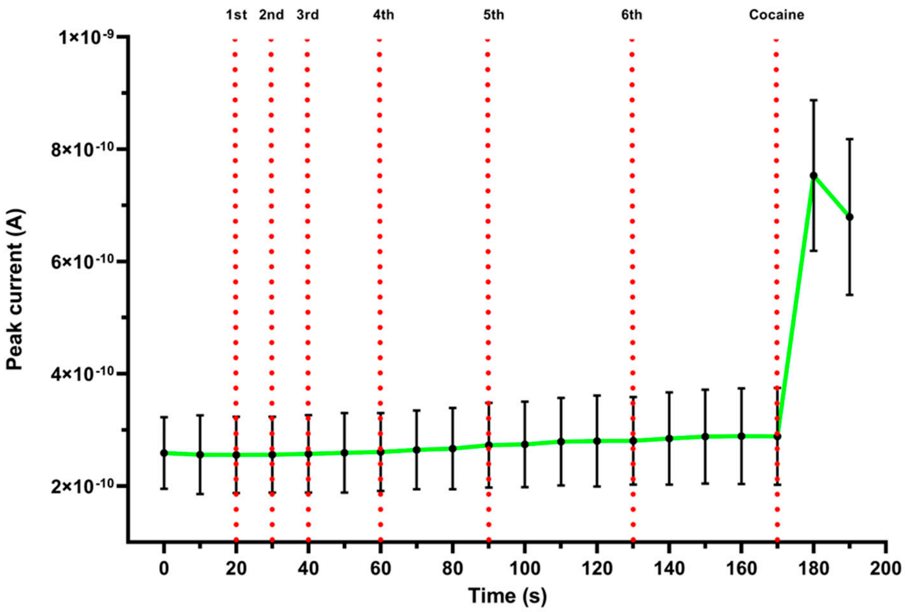

Figure S5. In vitro PBS injection experiment in 0.6% agarose. A 1μL PBS was delivered at different time points (20s, 30s, 40s, 60s, 90s, 130s) and 1μL 250mM cocaine solution was delivered at 170s. Red lines indicate timing of injections. Last injection is cocaine to confirm normal functionality of the sensor. No motion artifacts were observed after PBS injections and a clear response to cocaine was observed after the cocaine injection. N=6. Mean ± SEM.

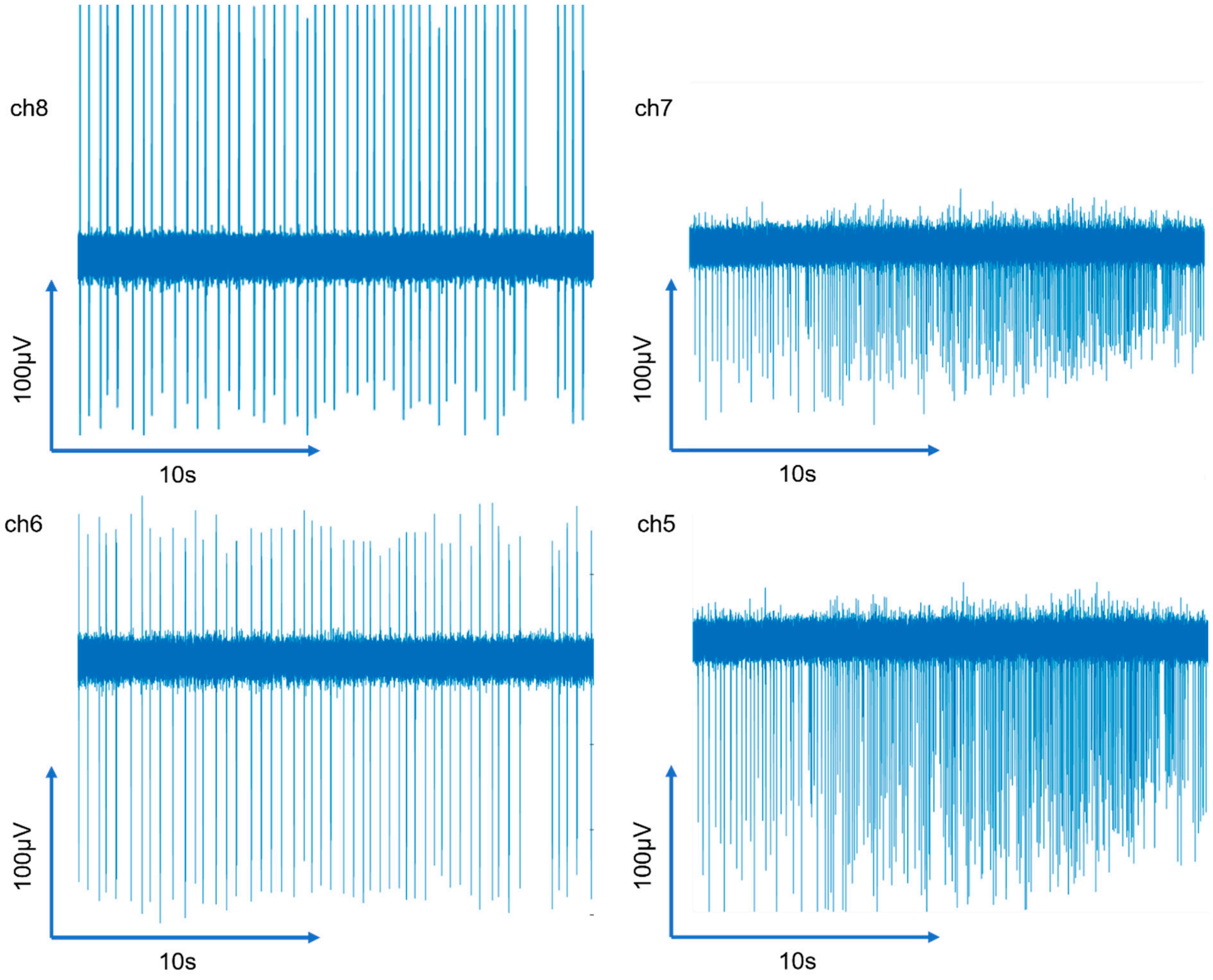

Figure S6. In vivo electrophysiology recording spike stream data. Filtered stream data plotted for corresponding representative units shown in Fig 6D. .

Table S1. Summary and comparison of previous works with PSB catechol zwitterionic polymer coatings.

	Polymer type	Surface types	Target Application	In vitro/in vivo
Yang et al. ref 69	PSB with catechol	hydrophobic electrospun poly (L-lactic) acid (PLLA) film	Non-specific Biomedical applications	In vitro only
Zhang et al. Ref 80	catechol and zwitterion bi-functionalized PEG	polydopamine-silver nanoclusters (PDA-Ag) nanoplatform functionalized glassy carbon electrodes	Aptasensor for adenosine triphosphate (ATP)	In vitro only
Li et al. ref 79	PSB with catechol and poly (carboxybetaine methacrylate) (pCBMA) with catechol	NH2, OH, and CH3 terminated self-assembled monolayers coated Au and bare Au.	Antifouling coatings for general applications in complex media.	In vitro only
Golabchi et al. ref 72	PSB with catechol stabilized with polydopamine	SiO_2	Implantable silicon based MEAs	In vitro<br>In vivo
This work	PSB with catechol	SU-8, and aptamer/6-mercaptohexanol functionalized fuzzy Au.	Flexible MEA for cocaine sensing and electrophysiology	In vitro<br>In vivo
